# Large Coronary Arteries Mean No Chance of a Heart Attack, Right? An Acute Myocardial Infarction in the Setting of Holding Anticoagulation for a Routine Colonoscopy

**DOI:** 10.7759/cureus.4544

**Published:** 2019-04-25

**Authors:** Nathan Brewster, Jennifer Nesfeder, Ryan Murphy, Brian Holahan, Syed Rafay Ali Sabzwari

**Affiliations:** 1 Internal Medicine, Lehigh Valley Health Network, Allentown, USA; 2 Cardiology, Lehigh Valley Health Network, Allentown, USA

**Keywords:** coronary artery ectasia, coronary ectasia, bridging therapy, cae

## Abstract

Coronary artery ectasia (CAE) is an uncommon pathology, which is sometimes incidentally found on left heart catheterization (LHC). CAE is occasionally treated with systemic anticoagulation to prevent thrombosis or progression of the clot in the coronary arteries. We present a 63-year-old male with known CAE on warfarin who presented to the hospital with myocardial infarction after a routine colonoscopy for which anticoagulation was held. His myocardial infarction was attributed to a likely coronary thromboembolic event. This case highlights the need for consideration of bridging anticoagulation therapy before and after procedures in patients with CAE to prevent adverse coronary events.

## Introduction

Coronary artery ectasia (CAE) is an uncommon pathology present in 1.4% to 4.9% of patients undergoing left heart catheterization. Multiple coronary artery involvement is even rarer [1-2]. The etiology of CAE includes atherosclerosis, Kawasaki disease, connective tissue disease, arteritis, and trauma [3-5]. Patients with CAE are often treated with antiplatelet therapy along with systemic anticoagulation in an effort to prevent thromboembolic events that could cause myocardial ischemia [1,6-8]. The effects of anticoagulation in these patients have not been studied extensively. We present a case of a patient with CAE who experienced a non-ST elevated myocardial infarction (NSTEMI) while off of anticoagulation after a colonoscopy.

## Case presentation

A 63-year-old male presented to the hospital with chest pain. His extensive cardiac history included multiple myocardial infarctions, severe triple vessel CAE, and recently a thrombus occluding flow through the second obtuse marginal artery. Due to the thrombus, he was initiated on warfarin to supplement the clopidogrel he was already taking. 

**Figure 1:**
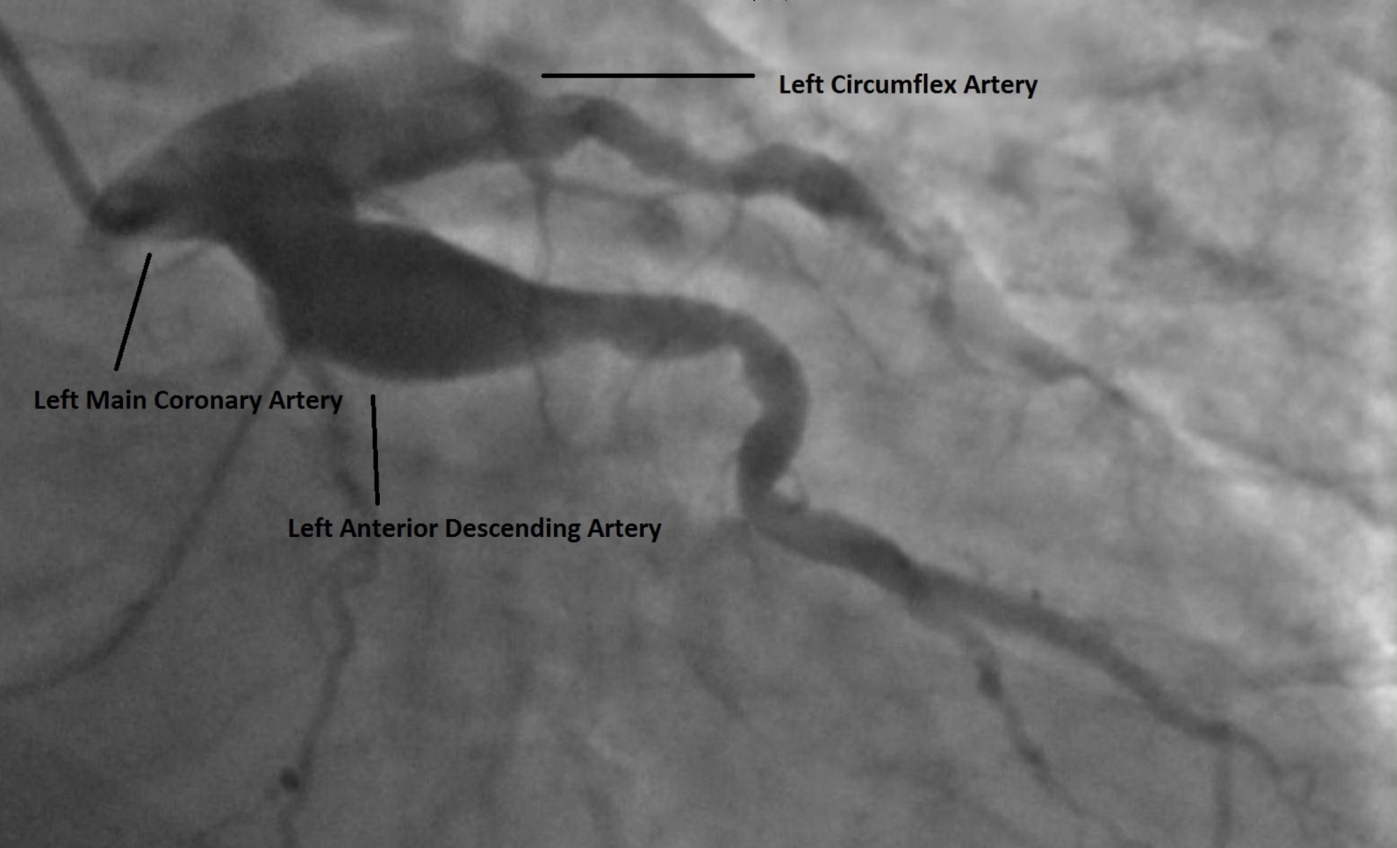
Ectasia of the left main, proximal left anterior descending, and proximal left circumflex coronary arteries

**Figure 2:**
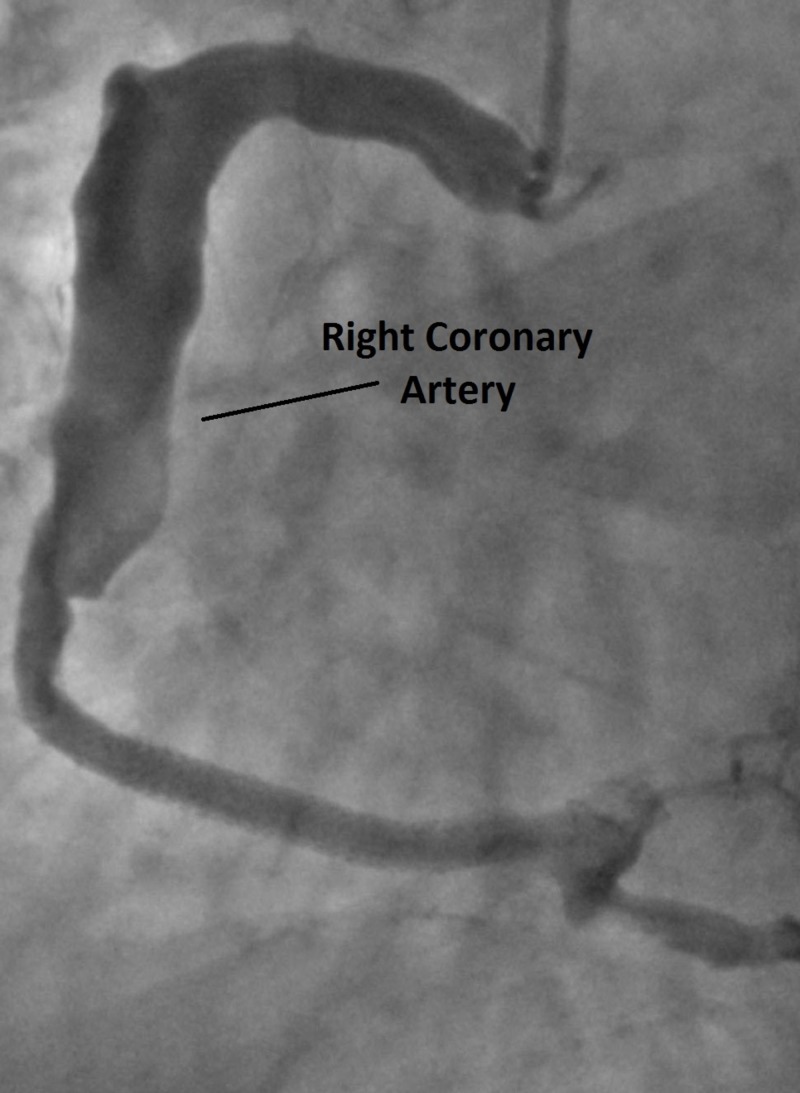
Ectasia of the right coronary artery

Preceding this hospitalization, the patient underwent a colonoscopy prior to which he discontinued his warfarin while being bridged with enoxaparin prior to the procedure. He had no complications up to and throughout the colonoscopy. As instructed, he resumed his warfarin but was not bridged post procedure. The following day, he developed chest pain radiating to his neck, diaphoresis, and dyspnea similar in presentation to his past myocardial infarctions. He arrived at the emergency department with no ST changes on electrocardiogram and was admitted to the hospital with a diagnosis of NSTEMI with a troponin peaking at 14.80 ng/mL. His INR was also subtherapeutic at 1.1. The patient was administered aspirin and clopidogrel and started on a heparin drip. Left heart catheterization showed severely ectatic vessels with a patent prior right coronary artery stent (Figures 1-2). There was no evidence of acute coronary occlusion. With medical management, the patient became chest pain and dyspnea free.

Due to the NSTEMI with transient chest pain and no new obstructive coronary artery disease, the patient was thought to have experienced a thromboembolic event involving the coronaries. He was restarted on warfarin while being bridged with enoxaparin injections until his INR was again therapeutic. There were no further events of chest pain, and resolution of troponin levels was seen. 

## Discussion

Patients on chronic anticoagulation with warfarin may benefit from bridging therapy to decrease the amount of time that patients are not anticoagulated to prevent adverse events. Bridging is generally considered beneficial in patients with a history of embolic stroke within the last three months, mechanical mitral valve, atrial fibrillation with a high CHA2DS2-VASc score, venous thromboembolism within the last three months, coronary stenting in the last 12 weeks, and previous thromboembolism during interruption of chronic anticoagulation [9]. 

Generally, anticoagulation post-procedure is considered on a case by case basis by determining the risks of post-procedure bleeding versus thrombotic events while not anticoagulated. When bleeding is a risk after a procedure due to performance of a biopsy or a surgical incision, it is usually considered safe to restart anticoagulation 48 to 72 hours after hemostasis has been achieved [10]. This can be even earlier in smaller scale interventions. 

For our patient, he did not have any increased risk of bleeding after his colonoscopy as he had no polypectomy or biopsy performed. Now that he has had an adverse embolic event while not being bridged, he should be bridged in the future. This patient also demonstrates the need to consider bridging therapy pre and post-procedure for those with CAE as the large vessel caliber is prone to blood stasis putting these patients at a higher risk of thrombus formation. This subset of patients has not been studied on a large scale and would benefit from further investigation.

## Conclusions

Patients with CAE may have an increased risk of thrombus formation because of the vessel's large caliber which is prone to blood stasis. These thrombi can then embolize into the distal vessel and cause ischemia. Although there is paucity of randomized trials, for those patients who are on warfarin for CAE, bridging therapy both before and after an elective procedure may be beneficial to prevent myocardial infarctions, especially in patients undergoing colonoscopies where no biopsies are performed and thus no increased risk of post-procedure bleeding.
